# A case report of anterior mediastinal angiomyolipoma that invaded the left thoracic cavity

**DOI:** 10.1097/MD.0000000000035786

**Published:** 2023-10-27

**Authors:** Dong Bai, Yuanzi Liang, Wenting Liu, Yanhan Liu, Zhiqun Wang

**Affiliations:** a Department of Radiology, Aerospace Center Hospital, Beijing, China; b Department of Pathology, Aerospace Center Hospital, Beijing, China.

**Keywords:** angiomyolipoma, computed tomography imaging, mediastinum

## Abstract

**Rationale:**

Angiomyolipoma is a mesenchymal tumor composed of blood vessels, smooth muscle, and mature adipose tissue. It is most commonly found in the kidney, and is rare outside the kidney, especially in the mediastinum. Only about 12 cases have been reported worldwide so far.

**Patient concerns:**

We report a young female patient who had been found with a left thoracic mass for 19 years. In the past 19 years, the patient had no chest pain, dyspnea and other symptoms, but this time she visited the doctor because of cough, and there were no other clinical signs.

**Diagnoses:**

The patient underwent computed tomography plain scan and enhanced scan after admission with imaging manifestations of a mixed density mass in the left chest cavity, calcification and fat density in the inside, and tortuous blood vessels after enhancement. Combined with imaging, the diagnosis was teratoma, not excluding hamartoma.

**Interventions:**

The patient underwent a central open thoracic giant mass resection.

**Outcomes:**

The postoperative pathology confirmed that it was angiomyolipoma originating from anterior mediastinum invasion of the left chest cavity, and no clear recurrence was seen after 1 year of postoperative follow-up.

**Lessons:**

Angiomyolipomas in the mediastinum are rare, especially those that invade the thorax. This article describes the clinical, imaging and pathological features of the patient in detail, which improves the understanding of the disease of clinical and imaging doctors, and provides a basis for the differential diagnosis of mediastinal lesions.

## 1. Introduction

Martignoni^[1]^ first described “angiomyolipomas” involving the kidney in 1951.Angiomyolipoma is a nonmalignant tumor that contains adipose tissue, smooth muscle cells, and blood vessels. The proportion of each ingredient may be variable. Angiomyolipoma is most common in the kidney, and extrarenal sites include the liver, nasal cavity, mediastinum, colon, lung, pancreas, vulva, and skin.^[2–6]^ So far, only about 12 cases of angiomyolipoma of mediastinum have been reported in the literature. This article reports a very rare case of anterior mediastinal angiomyolipoma that invaded the left thoracic cavity.

## 2. Case report

**Figure 1.**
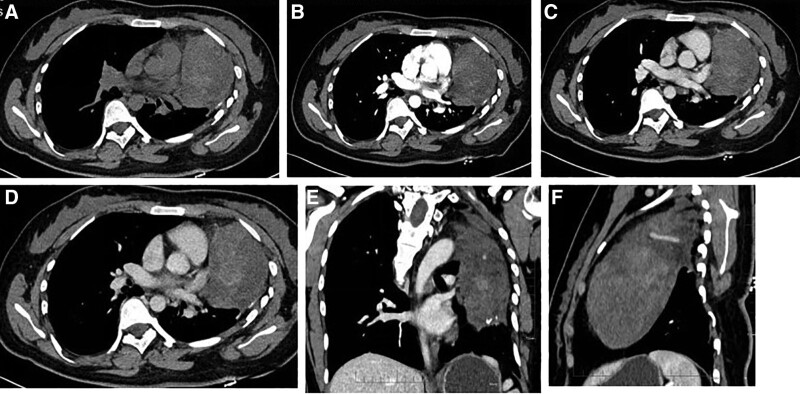
CT showed a mass of mixed density in the left thoracic cavity with calcification and fat density, and the boundary with the mediastinum was not clear. The enhancement was uneven, and the thick blood vessels were seen in the left thoracic cavity. CT = computed tomography.

A 24-year-old female patient presented to the local hospital 19 years ago with a “cough” and had a chest computed tomography (CT) examination showing a left chest mass. The patient had no chest tightness, shortness of breath, hemoptysis night sweats, no panic, no dyspnea, hoarseness or other discomforts. Postoperative pathology showed hamartoma and thymic cyst. The patient did not undergo special treatment, and regular reexamination showed no significant changes in the tumor. The patient coughed 1 month ago, and chest contrast-enhanced CT showed a smaller left thoracic cage. An irregular mixed density mass shadow was seen in the left chest cavity, about 6.4 cm × 9.0 cm × 13 cm in size, and the solid components of the enhanced scan showed mild to moderate sustained strengthening, and a thick draining vein could be seen in the lesion through the lesion and merged into the left brachiocephalic vein. There were many small flakes of fat density and calcification in it. The lesion margin was still smooth, and the local boundary from the adjacent mediastinum and chest wall tissue was not clear. Left pleural space, combined with history, mediastinal origin tumor, teratoma? Tumor of lung origin, hamartoma? The left lung was partially compressed (Fig. 1). The patient had no prior history of tuberous sclerosis. Physical examination showed thoracic symmetry without deformity, no tenderness, consistent bilateral respiratory movements, symmetrical bilateral tactile speech, clear sound on bilateral percussion, decreased breath sounds in the left lung, normal breath sounds in the right lung, no wet, and dry rales heard, or no pleural friction rub. Tumor marker examination was completed after admission, and the results were all negative.

**Figure 2.**
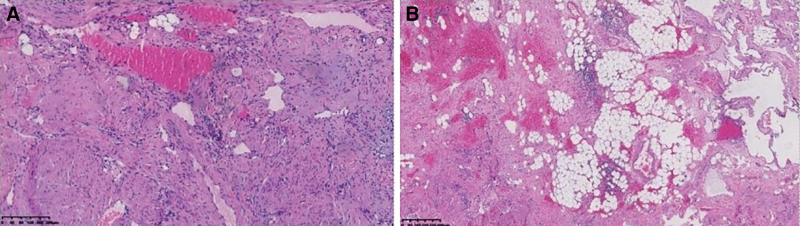
Pathology suggested hyperplastic smooth muscle tissue, thick-walled blood vessels and mature adipose tissue (HE staining, 100X, 40X).

The patient had surgical indications and no surgical contraindications, and planned to undergo thoracic tumor resection. Mediastinum and thorax were investigated: the tumor was located in the anterior mediastinum and invaded the left thorax. It was cystic, irregular in shape, without complete envelope, and rich in blood supply. The tumor was obviously adhered to the left lung, pericardium, left chest wall, and left innominate vein. Postoperative pathology: (left thoracic cavity) angioleiomyoma, total size 12 × 10 × 4 cm, found scattered fat, no necrosis, no significant cell atypia. Immunohistochemical results: Hematopoietic progenitor cell antigen CD34 (+), CD31 (+), anti-smooth muscle antibody (SMA) (+), Melanoma Antigen Melan-A (−), S-100 (focal +), Human melanoma antibody (HMB-45) (−), transcription factor e3 (−), Ki-67 (+ 1%), epidermal growth factor receptor (+) (Fig. 2). (Tumor envelope) was presented with atrophied thymus tissue. The patient was followed up for 1 year after surgery, and no clear signs of recurrence were found. The Hospital Ethics Review Board approved it, and the patient was informed about and consented to enter the study.

## 3. Discussion

Angiomyolipoma used to be considered hamartoma or chorea, but is now considered a tumor. Evidence of neoplasm includes monoclonality and the presence of multiple cytogenetic abnormalities. Perivascular epithelioid cells are thought to be cells of origin for this and other related tumors and tumor-like processes.^[7]^Perivascular epithelioid cell tumors are mesenchymal tumors included in the 2002 World Health Organization Soft Tissue Classification, characterized by organ-like, radial arrangement of epithelioid cells around blood vessels and specific expression of melanocytes and myocyte markers. Angiomyolipoma is the most common pathological type in the perivascular epithelioid cell tumors family, often involving the kidneys and liver, but also in other sites, such as the heart, mediastinum, lungs, palate, vagina, etc.^[8]^ However, only about 12 cases of mediastinal lesions have been reported in the literature, ranging from 22 to 69 years of age, with an average age of about 45 years, with a male to female ratio of about 1:1, occurring in 50% of anterior mediastinal lesions, and only 1 case with nodular sclerosis. Most of the patients had no obvious symptoms, small lesions and clear boundaries. In this case, the left chest space was found due to cough when he was a child, and he was followed up for the next 19 years without obvious symptoms. This time, he visited the hospital due to cough, and the breath sounds of the left lung were weakened. The clinical symptoms were consistent with the literature reports.

The pathogenesis of the angiomyolipoma is unclear, but in recent years it has been suggested that angiomyolipoma is closely related to tuberous sclerosis.^[9]^Barnes et al^[10]^ speculated on the pathogenesis of lymphangioleiomyomatosis and angiomyolipoma which the deletion of the tuberous sclerosis 2 gene in neural crest epithelial cells may potentially promote epithelium-to-mesenchymal transition, leading to invasion and migration of mutant cells to distant sites such as the lungs, kidneys, and axial lymphatic vessels and tumorigenesis.

The preoperative diagnosis of angiomyolipoma (AML) mainly relies on imaging and tumor biopsy. Currently, there are few reports on angiomyolipoma images. The imaging findings are closely related to their tissue composition, and the distribution of blood vessels and mature adipose tissue are the most important diagnostic features. According to the different proportions of internal blood vessels, smooth muscle, and fat components, AML is divided into 4 subtypes.^[11]^The first is mixed type, more common, blood vessels, fat cells, and smooth muscle cells accounted for a certain proportion or a similar proportion of mixed distribution. CT plain scan shows a mixed density containing fat, and some calcification could be seen. Enhanced scan shows continuous enhancement of the lesions, and high enhanced vascular shadows could be seen in the center or edge of the lesions. The second is leiomyoma type, CT enhanced scan mostly shows continuous enhancement, but some lesions could not be accurately diagnosed before surgery because it is difficult to find typical fat density on CT. The 3rd is lipoma type, rich fat is its characteristic, other components are relatively few. The 4th is hemangiomatous type (rich blood vessels), the main component is large, deformed, distorted blood vessels, less cell components, and the strengthening way is similar to hemangioma. The adipose tissue in AML shows significantly low density on CT plain scan (about −120 to −60 HU), and the signal of MRI adipose inhibition sequence is significantly reduced. At present, the value of PET-CT in the diagnosis of AML is unclear, and PET/CT in AML mostly shows a slightly higher, equal or slightly lower uptake of 18F-FDG, with no obvious specificity. The CT findings of the patient in this case showed an irregular mixed density mass with a size of about 6.4 cm × 9.0 cm × 13 cm in the left thoracic cavity. The solid components of the enhanced scan showed mild to moderate continuous enhancement, and a large drainage vein could be seen in the lesion passing through the lesion and merging into the left brachial vein. There are many small flaky fat density shadows and calcification shadows. The lesion had smooth edges and indistinct local demarcation with adjacent mediastinum and chest wall tissue. Fat density and thick vascular shadows were seen in the lesions. The imaging findings were consistent with those reported in the literature, and the lesions were considered to be mixed type, with a certain proportion of blood vessels, fat cells and smooth muscle cells. However, the original image positioning in this paper considered the left thoracic cavity, but the intraoperative manifestation was anterior mediastinal lesion invading the left thoracic cavity. Therefore, follow-up image analysis showed that the lesion was located in the left thoracic cavity. However, careful analysis found that the upper edge of the lesion was connected to the adipose space of the anterior mediastinum, the lesion margin was relatively clear, and it was at an acute angle to the left chest wall, and the adjacent lung tissue was ataxially changed. Follow-up imaging showed that the lesion was located in the mediastinum and invaded the chest cavity.

Angiomyolipoma is composed of smooth muscle cells, blood vessels and mature fat cells. Blood vessel components are usually zigzag and thick-walled, have lower elastin content than normal blood vessels, and are usually surrounded by smooth muscle cells. The lack of elastic tissue in tumor blood vessels predisposes patients to aneurysms and spontaneous bleeding. Smooth muscle appears as normal spindle or round epithelioid cells, which vary in number from the area near blood vessels to the main body of the tumor. Smooth muscle nuclei are usually small and regular, but some regions occasionally have enlarged atypical nuclei, nucleolus, and rare mitosis. Immunochemically, HMB-45 is consistently expressed in angiomyolipomas. Other markers that are commonly positive in angiomyolipoma include SMA, CD34, and CD-68.^[12,13]^ The most important cells used for diagnosis are smooth muscle cells, the smooth muscle components often originate in the blood vessel wall and may appear cellular, atypical, pleomorphic, or epithelioid. The postoperative pathology of this patient was angiomyolipoma, with thick-walled blood vessels, proliferative smooth muscle tissue and mature fat, no necrosis, and no significant cell atypia. Immunohistochemical results were CD34 (+), CD31 (+), SMA (+), Melan-A (−), S-100 (focus +), HMB-45 (−), transcription factor e3 (−), Ki-67 (+ 1%), epidermal growth factor receptor (+).

The pathological findings in this study were consistent with angiomyolipoma, but different from previous literature, the immunohistochemical index HMB-45 was negative. It has been reported that angiomyolipoma is found differently in the head and neck area than in the kidney and liver because HMB-45 immunohistochemical staining is negative. As in our case, microscopic examination showed no epithelioid cells and HMB-45 staining of smooth muscle cells was negative. However, as with the skin site, these cases were not associated with tuberous sclerosis and did not express HMB-45, which led to the latter being classified as mucocutaneous angiomyolipoma.^[4,14]^The pathogenesis of HMB-45 expression remains unclear. Assumptions have been made in previous reports. First, HMB-45 expression in angiomyolipoma may be associated with nodular sclerosis. Second, HMB-45 expression may help distinguish angiomyolipomas with epithelioid components from other tumors. But it may not be helpful in distinguishing angiomyolipomas without epithelioid components from other tumors. Third, fat percentage may affect HPB-45 because clear cells may represent perivascular epithelioid cells that are undergoing lipid degeneration.^[15,16]^Further studies are needed to consider the expression of HMB-45 in angiomyolipoma. A small percentage of AML does not express HMB-45 or focally expresses HMB-45, while PNL2 is a molecular marker of epithelioid melanoma, and its specificity and sensitivity for the diagnosis of AML are higher than HMB-45, which can better reflect the cell source.^[17]^

On imaging, angiomyolipomas that occur in the mediastinum and invade the thorax need to be differentiated from diseases with fatty components in the mediastinum and thorax. First of all, the teratoma is composed of 2 to 3 embryonic developmental tissues, which can be seen as fat density and calcification as angiomyolipoma on imaging. However, the teratoma is mainly cystic, with uneven sac wall thickness, visible separation, and enhancement after enhancement, but there is no obvious tortuous and thickened blood vessels. Secondly, mediastinal lipomas are uncommon and benign mesenchymal tumors. It is more common in the anterior mediastinum and the cardiophrenic angle area, with smooth edges, clear boundaries with surrounding structures, and often a thin and uniform envelope. On enhanced scan, the tumor parenchyma was not enhanced, the envelope could be slightly enhanced, and the density was relatively uniform to fat density. Finally, The imaging findings of liposarcoma are an area of solid density with uneven and enhanced density, unclear boundary and obvious peripheral infiltration. Angiomyolipoma has a clear boundary, and the surrounding tortuous and thickened vessels can be seen by enhancement, so it can be distinguished.

In terms of treatment, usually asymptomatic angiomyolipomas may not usually require intervention. Imaging tests may be repeated annually or semiannually to assess disease stability and progression. Indications for intervention include suspected malignancy, spontaneous bleeding with obvious symptoms, pain, hematuria or risk of rupture or other complications. Treatment depends on location, clinical presentation and the site and size of the growth. Complete resection of the tumor is the ideal form of treatment. At present, more and more institutions choose vascular embolization, which can not only stop bleeding quickly, but also make the lesion smaller and not easy to relapse. Our case responded well to surgical resection and did not have any recurrence for up to 1 year.

The limitations of this paper are that this patient did not undergo chest MR Examination, so the MR Manifestations of mediastinal angiomyolipoma invading the thoracic cavity need to be improved. In addition, the previous imaging data of the patient was not obtained, so it could not be compared, and it is hoped that it can be improved in the future.

## 4. Conclusion

We presented a case of angiomyolipoma invading the left thoracic cavity. CT mainly showed a jumbled density of space containing fat and calcification. After enhancement, large tortuous vessels could be seen, which had certain imaging characteristics, but immunohistochemical HMB-45 was negative. It provides some value for the diagnosis of angiomyolipoma in the future.

## Author contributions

**Data curation:** Yuanzi Liang.

**Investigation:** Wenting Liu.

**Resources:** Yanhan Liu.

**Writing – original draft:** Dong Bai.

**Writing – review & editing:** Zhiqun Wang.
